# The unaided recovery of marathon-induced serum metabolome alterations

**DOI:** 10.1038/s41598-020-67884-9

**Published:** 2020-07-06

**Authors:** Zinandré Stander, Laneke Luies, Lodewyk J. Mienie, Mari Van Reenen, Glyn Howatson, Karen M. Keane, Tom Clifford, Emma J. Stevenson, Du Toit Loots

**Affiliations:** 1 Human Metabolomics, Faculty of Natural and Agricultural Sciences, North-West University, Potchefstroom, 2531 South Africa; 2 Department of Sport, Exercise and Rehabilitation, Faculty of Health and Life Sciences, Northumbria University, Newcastle upon Tyne, NE1 8ST UK; 3 Water Research Group, School of Environmental Sciences and Development, North-West University, Potchefstroom, 2531 South Africa; 4 Human Nutrition Research Centre, Institute of Cellular Medicine, Newcastle University, Newcastle upon Tyne, UK; 5 School of Sport, Exercise and Health Sciences, Loughborough University, Loughborough, UK; 6 North-West University, Private Bag X6001, Box 269, Potchefstroom CampusPotchefstroom, 2520 South Africa

**Keywords:** Metabolomics, Metabolomics

## Abstract

Endurance athlete performance is greatly dependent on sufficient post-race system recovery, as endurance races have substantial physiological, immunological and metabolic effects on these athletes. To date, the effects of numerous recovery modalities have been investigated, however, very limited literature exists pertaining to metabolic recovery of athletes after endurance races without the utilisation of recovery modalities. As such, this investigation is aimed at identifying the metabolic recovery trend of athletes within 48 h after a marathon. Serum samples of 16 athletes collected 24 h before, immediately after, as well as 24 h and 48 h post-marathon were analysed using an untargeted two-dimensional gas chromatography time-of-flight mass spectrometry metabolomics approach. The metabolic profiles of these comparative time-points indicated a metabolic shift from the overall post-marathon perturbed state back to the pre-marathon metabolic state during the recovery period. Statistical analyses of the data identified 61 significantly altered metabolites including amino acids, fatty acids, tricarboxylic acid cycle, carbohydrates and associated intermediates. These intermediates recovered to pre-marathon related concentrations within 24 h post-marathon, except for xylose which only recovered within 48 h. Furthermore, fluctuations in cholesterol and pyrimidine intermediates indicated the activation of alternative recovery mechanisms. Metabolic recovery of the athletes was attained within 48 h post-marathon, most likely due to reduced need for fuel substrate catabolism. This may result in the activation of glycogenesis, uridine-dependent nucleotide synthesis, protein synthesis, and the inactivation of cellular autophagy. These results may be beneficial in identifying more efficient, targeted recovery approaches to improve athletic performance.

## Introduction

Endurance races (> 5 km) have become increasingly popular over the last decade and primarily include half-marathons (21.1 km), marathons (42.2 km), and ultra-marathons (> 42.2 km)^1^. Participation in these events requires extensive preparation including physical and mental conditioning as well as meticulously planned dietary strategies. According to Barnett^2^, optimal athletic performance is only achieved when these principles are accompanied by sufficient system recovery following any endurance activity. “Recovery” comprehensively refers to the process in which an altered biological system, such as the metabolome, reverts to its corresponding pre-perturbed state^3^. In endurance athletes, metabolic recovery is thought to proceed in a biphasic manner with the initial phase mainly consisting of rapid oxygen, ATP and phosphocreatine replenishment, whereas the second phase entails the slow restoration of innate metabolism adaptations^2,3^. A popular research approach for identifying and mapping such metabolic adaptations, quantitatively and qualitatively, is collectively referred to as “metabolomics”. Considering that the metabolome provides a direct depiction of the physiological state of an organism at a specific point in time^4^, metabolomics is a formidable tool when investigating the effects of endurance races on the human body, as well as the recovery thereof.

Previous studies investigating the perturbed metabolism of endurance athletes mainly observed elevated concentrations of carbohydrates, fatty acids, and ketones, accompanied by reduced concentrations of amino acids^5^. This is proposed to result from the dramatic metabolic shift between numerous fuel substrate catabolism systems, as well as alternative energy-producing mechanisms (α-oxidation and autophagy) activated during strenuous activity^6–10^. Additionally, the extensive reduction in fuel substrates was associated with alterations in the urea cycle, purine, steroid and pyrimidine metabolism, reactive oxygen species production pathways^5,6,9–13^, as well as the gut microbiome^9,14^.

Given the nature and extent of the physiological, immunological and metabolic effects of endurance exercise on the human body, a substantial amount of research has been directed towards investigating various therapeutic recovery modalities including cryotherapy, massage, heat therapy, stretching, compression, and non-steroidal anti-inflammatory drugs ^2,15–17^, as well as the ingestion of functional foods, such as cherries, pomegranates, and green leafy vegetables^18^. However, very limited literature is available regarding the overall recovery of the perturbed metabolism after an endurance race, without the ingestion and/or utilisation of recovery modalities. From these studies, it is evident that during the immediate hours after recovery, myocellular glycogen restoration^19,20^, as well as phosphocreatine store replenishment and blood lactate removal takes preference^2,3,21^, while lipids are temporarily further oxidised as an energy source^19,20^. Considering this, it is suggested that glycogen restoration is attained within 24–48 h after endurance exercise^20^, whereas amino acids, fatty acids, fatty acid-carnitine conjugates along with tricarboxylic acid (TCA) cycle and pyrimidine/purine pathway intermediates remain perturbed for at least 14 h after endurance running^5–7,9^.

Considering the current lack of literature regarding the metabolic recovery of marathon athletes over an extended period (48 h), an untargeted two-dimensional gas chromatography time-of-flight mass spectrometry (GCxGC-TOFMS) serum metabolomics approach was used to investigate the unaided (without the ingestion and/or utilisation of recovery modalities) metabolic recovery (to baseline-related concentrations) of 16 marathon athletes within 48 h post-marathon. These results might improve the current knowledge pertaining to the consequence of endurance activity and the time-course of recovery in the days following the event, as well as lead to the identification of new or additional recovery pathways and/or more targeted, perhaps even personalised, recovery approaches.

## Results

**Figure 1:**
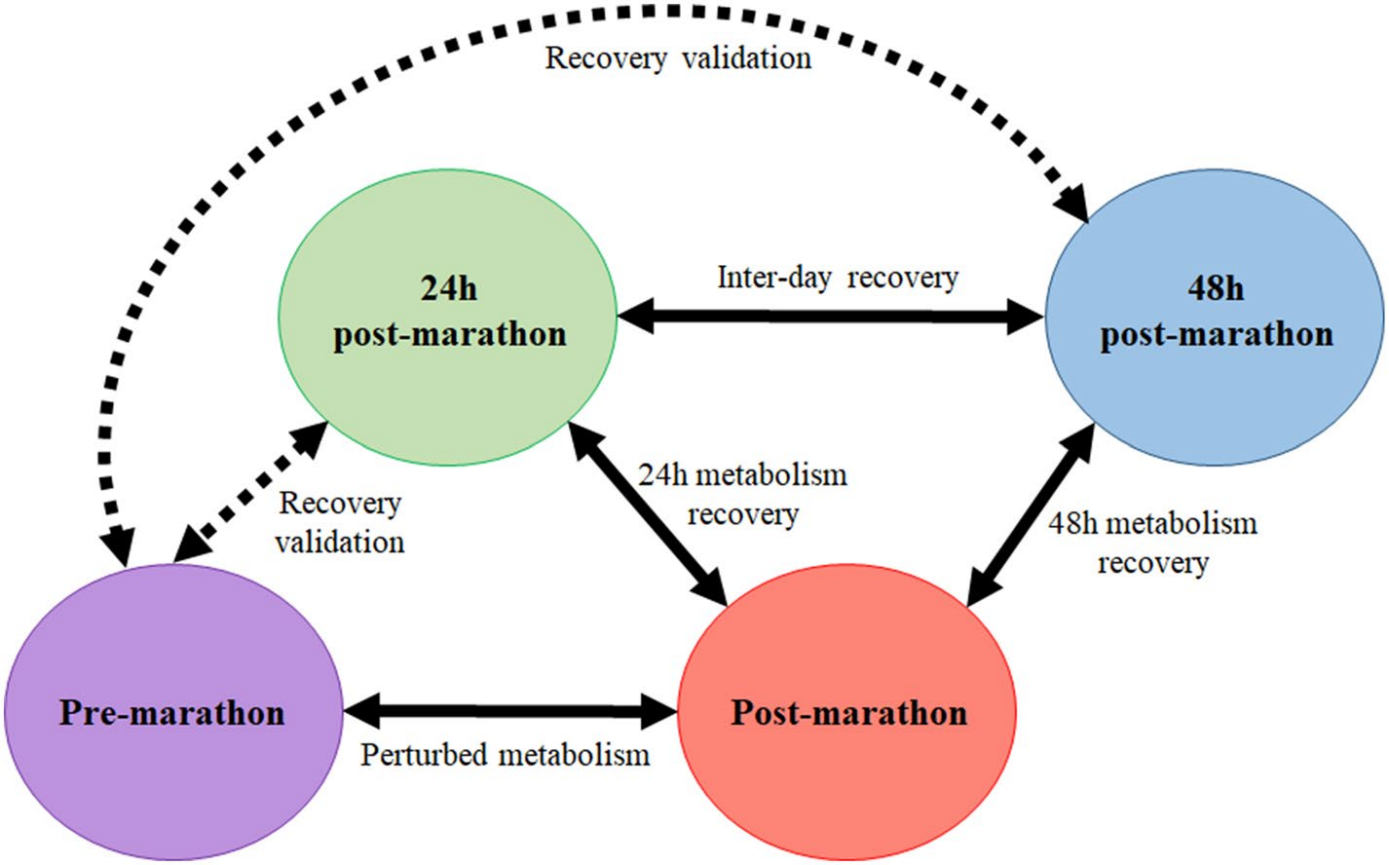
Schematic representation of the sample group comparisons performed during this investigation.

The GCxGC-TOFMS system detected a total of 1,308 peaks of which 444 peaks were classified as “Analytes/Unknowns”. After removing column and/or reagent-related compounds, as well as following the merging of duplicate peaks of compounds, such as sugars and amino acids, 839 compounds (including the 444 peaks classified as “Analytes/Unknowns” and internal standard) remained. The entire list of 838 compounds (excluding internal standard) was then subjected to the aforementioned processing steps [i.e. quality control coefficient of variation (QC-CV) filter etc.] and further statistical analyses. Considering the aim of this investigation, multiple group comparisons were required (Fig. 1), subsequently leading to numerous statistical outputs, which will be discussed below.

### Multivariate statistical output

**Figure 2:**
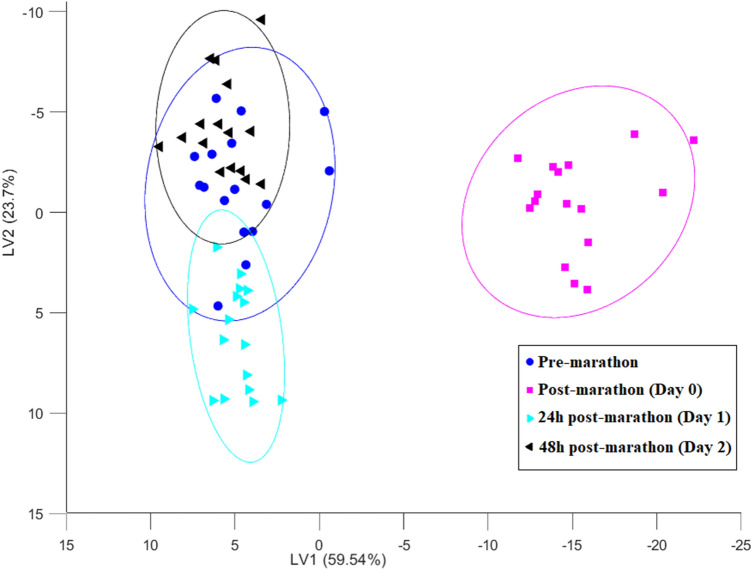
The ASCA plot indicating the natural, time-dependent differentiation of the comparative groups: pre-marathon (denoted by blue/circle), post-marathon (denoted by pink/square), 24 h post-marathon (denoted by turquoise/right-tilted triangle) and 48 h post-marathon (denoted with black/left-tilted triangle). The variance accounted for by each latent variable (LV) is indicated in parenthesis on the respective axes. The ellipsoids represent 95% confidence intervals of each time-points centroid.

In order to visualise the overall metabolic shift over the entire timeframe of the study, an analysis of variance (ANOVA) simultaneous component analysis (ASCA) model was constructed with the resulting scores graphically displayed and grouped according to the time component of the current study design (Fig. 2). All scripts used here are readily available in the mentioned toolboxes provided by Eigenvector Research Inc., with the exception of the ASCA scripts, which can be downloaded from https://www.bdagroup.nl/content/Downloads/software/software.php. Based on Fig. 2, it is evident that although a marathon has a dramatic impact on the human serum metabolome these changes recover within 48 h, as indicated by the confined positioning of the 24 h and 48 h post-marathon ellipsoids relative to that of the pre-marathon. Furthermore, in an attempt to inspect specific adaptations between time-points, individual multi-level principal component analysis (ML-PCA) plots for the specified group comparisons (Fig. 1) are presented in Supplementary Fig. S1a–f. The following can be deduced from these figures (S1a–f): (1) As expected, the marathon perturbation (Fig. S1a) induced significant metabolic alterations, which is supported by Stander et al.^10^; (2) Clear separation (no overlap) of the recovery time-points (24 h and 48 h post-marathon) and post-marathon (day 0) ellipsoids (Fig. S1b and c) indicate a significant deviation from the post-marathon perturbed metabolic state; (3) The magnitude of metabolome variation presented 24 h and 48 h post-marathon (Fig. S1 f) seems rather minor as these ellipsoids appear relatively grouped (ellipsoid overlap); (4) The confined positioning of the 24 h and 48 h post-marathon ellipsoids relative to the pre-marathon (Fig. S1 d and e) indicate the recovery of the metabolome to a baseline-related state (overlapping ellipsoids). Furthermore, in order to visualise metabolite stratification among the comparative groups, clustering dendrograms were constructed and are included in the supplementary material (Fig. S3). From these figures, it is clear that stratification associations did not correlate with the comparative groups.

However, considering the relatively small sample set and the likelihood of increased false discovery rates (FDR; high dimensionality), along with controlling for model overfitting, multivariate models could not be validated and were therefore not used for variable selection but rather for global metabolic shift visualisation.

### Univariate statistical outputs

Considering the aforementioned, variable selection was based on univariate comparisons as a means of determining the specific metabolic adaptions apparent during the course of this investigation while controlling the FDRs and avoiding overfitting. Although repeated measures ANOVA models may have been appropriate, paired t-tests were preferred since the dramatic effect of the marathon would result in a large number of significant metabolites requiring pairwise comparisons as post-hoc tests in any event. Based on this, only metabolites with a Benjamini–Hochberg *p *value ≤ 0.05 and an effect size *d*-value ≥ 0.5 (moderate effect) in each group comparison (Fig. 1), were deemed to be statistically significant. This approach yielded a list of 74 metabolite markers, of which 61 were positively annotated (the remaining 13 metabolites were classified as “Unknowns/Analytes”) and are listed in Table 1. A more comprehensive table including average concentrations at each of the comparative time-points has been included in Supplementary Table S1.

**Table 1:** Ratio of concentration fluctuations in the serum metabolite markers (n = 61) best describing the detected variation and metabolic recovery of the athletes within 48 h post-marathon.

Compound name (PubChem ID)	PM/PrM ratio	D1/PM ratio	D2/PM ratio	D2/D1 ratio	D1/PrM ratio	D2/PrM ratio
**Carbohydrate and associated intermediates**						
Arabitol (439255)	1.36*	0.75*	0.65*	0.87	1.01	0.88
Erythritol (222285)	1.77*	0.61*	0.39*	0.63	1.08	0.68
Glucose (5793)	8.91*	0.17*	0.09*	0.52	1.48	0.77
Mannitol (6251)	1.34	0.78	0.58*	0.75	1.05	0.78
Mannose (18950)	15.72*	0.11*	0.05*	0.49	1.66	0.82
Myo-inositol (440194)	1.10	0.76*	0.63*	0.82	0.84	0.69
Psicose (441036)	1.15	0.45*	0.70	1.56	0.52	0.80
Rhamnose (5460029)	0.62*	1.51	1.86*	1.23	0.94	1.16
Ribose (10975657)	1.16	0.58*	0.89	1.53	0.67	1.03
Sorbose (439192)	1.46	0.69	0.64*	0.94	1.01	0.94
Tagatose (439312)	1.87*	0.56*	0.53*	0.95	1.04	0.99
Talose (441035)	1.69*	0.57*	0.52*	0.91	0.96	0.87
Threonic acid (151152)	1.39*	0.80*	0.78*	0.98	1.11	1.09
Uridine (6029)	1.06	0.61*	1.02	1.66*	0.65	1.08
Xylose (135191)	0.90	0.52*	0.76	1.45	0.47*	0.69
**Lipid and associated intermediates**						
α-Hydroxyoctanoic acid (94180)	1.49*	0.63*	0.67	1.06	0.94	0.99
α-Linolenic acid (5280934)	1.26*	0.69*	0.74*	1.07	0.87	0.93
β-Hydroxyhexanoic acid (11829482)	3.00*	0.38*	0.31*	0.82	1.13	0.92
5-Dodecenoic acid (5312377)	35.22*	0.02*	0.06*	3.73	0.59	2.19
10-Heptadecenoic acid (5312434)	5.87*	0.12*	0.15*	1.22	0.73	0.89
11,14-Eicosadienoic acid (5282805)	1.94*	0.54*	0.45*	0.83	1.05	0.87
11-Eicosenoic acid (5282768)	3.48*	0.31*	0.37*	1.20	1.06	1.28
Docosahexaenoic acid (445580)	1.17	0.69*	0.80	1.17	0.80	0.94
Eicosapentaenoic acid (446284)	1.30	0.69*	0.74*	1.07	0.90	0.96
Glycerol (753)	5.84*	0.17*	0.19*	1.11	0.99	1.10
Glycerol monopalmitic acid (308463)	1.87*	0.44*	0.55*	1.24	0.82	1.02
Heptadecanoic acid (10465)	1.82*	0.52*	0.52*	1.01	0.94	0.95
Lauric acid (3893)	2.07*	0.57*	0.46*	0.81	1.17	0.95
Methyl oleic acid (9922235)	1.68*	0.48*	0.72	1.51	0.81	1.22
Myristoleic acid (5281119)	12.33*	0.06*	0.08*	1.32	0.70	0.93
Oleic acid (445639)	5.04*	0.21*	0.22*	1.07	1.06	1.13
Palmitic acid (985)	1.67*	0.55*	0.57*	1.04	0.93	0.96
Palmitoleic acid (445638)	6.26*	0.19*	0.22	1.17	1.20	1.40
Pentadecanoic acid (13849)	2.21*	0.51*	0.43*	0.85	1.13	0.96
**Ketones and TCA cycle intermediates**						
α-Ketoglutaric acid (51)	1.57*	0.58*	0.64*	1.11	0.91	1.01
β-Hydroxybutyric acid (441)	12.98*	0.06*	0.06*	1.12	0.72	0.81
Acetoacetic acid (96)	2.89	0.37*	0.19*	0.51	1.07	0.54
Citric acid (311)	1.46	0.74	0.58*	0.78	1.08	0.85
Fumaric acid (444972)	1.42	0.75	0.68*	0.91	1.06	0.96
Malic acid (525)	1.62*	0.57*	0.52*	0.90	0.93	0.84
Malonic acid (867)	1.37	0.72	0.62*	0.86	0.99	0.85
**Amino acid and cholesterol-associated intermediates**						
α-Ethylhydracrylic acid (188979)	2.24*	0.48*	0.47*	0.97	1.08	1.05
α-Hydroxybutyric acid (11266)	2.53*	0.52*	0.49*	0.93	1.31	1.23
β-Hydroxyisobutyric acid (87)	15.39*	0.04*	0.06*	1.29	0.67	0.87
β-Hydroxyisovaleric acid (69362)	1.15	0.89	0.75*	0.84	1.02	0.86
5-Pregnen-3β,20α-diol (312224064)	1.93*	0.38*	0.36*	0.96	0.73	0.70
Alanine (5950)	0.64	2.34*	1.86*	0.79	1.51	1.19
Aminomalonic acid (100714)	0.48	2.10*	2.06*	0.98	1.01	0.99
Aspartic acid (5960)	0.60	2.03*	1.36	0.67	1.22	0.82
Glutamic acid (33032)	0.79	1.51*	1.39	0.92	1.19	1.10
Glutaric acid (743)	1.73	1.12	0.52*	0.46	1.94	0.89
Glycine (750)	0.59	1.79*	1.45	0.81	1.06	0.86
Hydroxyproline (5810)	0.44	2.03	2.68*	1.32	0.89	1.18
Methionine (6137)	0.54	2.17*	1.88*	0.87	1.18	1.02
Phenylalanine (6140)	0.64	1.93*	1.47*	0.76	1.24	0.95
*p-*Hydroxyphenylacetic acid (127)	2.00	0.51*	0.45*	0.88	1.03	0.90
*p*-Hydroxyphenyllactic acid (9378)	1.53	0.66*	0.58*	0.88	1.01	0.89
Pyroglutamic acid (7405)	0.71*	1.35*	1.41*	1.04	0.95	1.00
Squalene (638072)	3.39*	0.31*	0.34*	1.07	1.06	1.14
Tyrosine (6057)	0.64	1.78*	1.57*	0.88	1.14	1.01
Valine (6287)	0.53	1.83	2.21*	1.21	0.97	1.18

*PrM* Pre-marathon, *PM* Post-marathon, *D1* 24 h post-marathon, *D2* 48 h post-marathon.

Significant *p*-and *d*-values (*).

## Discussion

Table 1 depicts the recovery trend of the metabolites not only initially affected by the marathon perturbation, but also the concentration fluctuations of additional metabolites pertinent to the 24 h and 48 h recovery period. In order to ascertain the metabolic recovery mechanisms following a marathon, it is imperative to first consider the immediate effects of a marathon on the serum metabolome of athletes, which was characterised and previously described by Stander et al.^10^. Briefly, alterations were mainly observed in metabolic pathways associated with energy production, such as elevated concentrations of various carbohydrates, TCA cycle intermediates, fatty acids and ketone bodies, and reduced concentrations of various amino acids and associated metabolites. The perturbed metabolic state may also be linked to imbalanced redox potential, the saturation of traditional fatty acid catabolic pathways, possible insulin secretion hampering, and the activation of cellular autophagy to provide additional fuel substrates.

**Figure 3:**
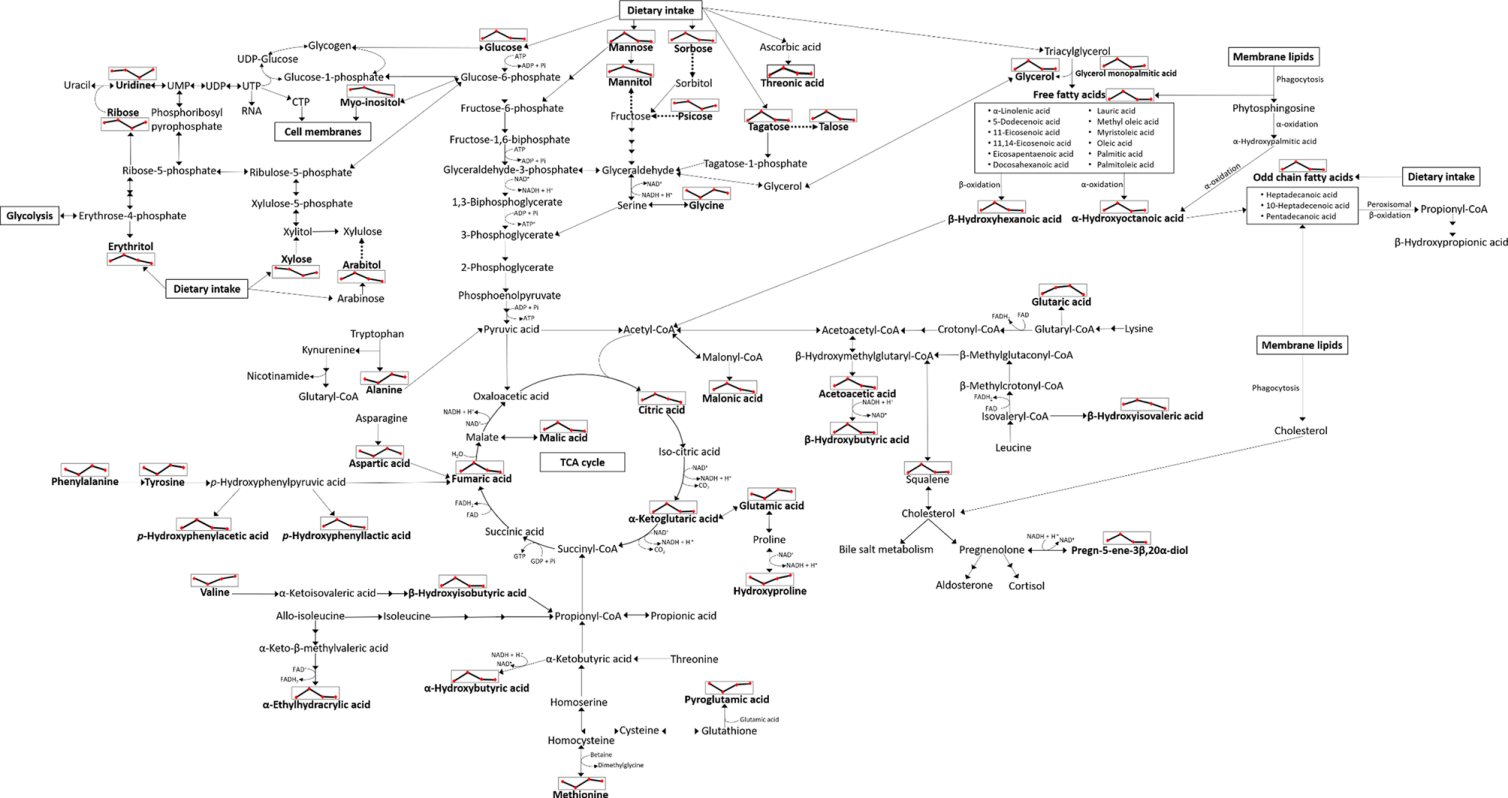
A metabolic chart summarising the major pathways affected during and after a marathon perturbation. Microbial interactions are denoted with bold dashed arrows and the line-graphs schematically illustrate the time-dependant concentration shifts of the significantly altered metabolites. *CoA* coenzyme A, *CO*_*2*_ carbon dioxide, *CTP* cytidine triphosphate, *FAD*^+^ flavin adenine dinucleotide, *FADH*_*2*_ flavin adenine dinucleotide + hydrogen, *GDP* guanosine diphosphate, *GTP* guanosine triphosphate, *NAD*^+^ nicotinamide adenine dinucleotide, *NADH* nicotinamide adenine dinucleotide + hydrogen, *Pi* inorganic phosphate, *TCA* tricarboxylic acid, *RNA* ribonucleic acid, *UDP* uridine diphosphate, *UMP* uridine monophosphate, *UTP* uridine triphosphate (adapted from Stander et al.^10^).

A detailed description of the recovery (to pre-marathon related concentrations) of the identified metabolites are discussed in a grouped-manner, based on metabolic alterations of the recovery time-points (24 h and 48 h post-marathon; day 1 and day 2) relative to the perturbed metabolic state (day 0; immediately post-marathon) and is schematically summarised in Fig. 3.

### Carbohydrate metabolism

Carbohydrates, glucose in particular, are considered to be the primary fuel substrates used by the body during physical activity. Stander et al.^10^ and Lewis et al.^6^ reported severely elevated serum concentrations of various carbohydrates immediately after a marathon (day 0). In this metabolomics investigation, it is clear that the majority of the serum carbohydrates (glucose and mannose) and associated metabolites (uridine, ribose, glycerol, and myo-inositol) returned to baseline (pre-marathon) concentrations within 24 h post-marathon (Table 1), with the exception of xylose (also dietary-related). This can be ascribed to the restoration of the glycogen stores, which were perceived as "depleted" in the initial stages of the marathon^10^.

Moreover, substantial reductions in uridine concentrations were observed within 24 h post-marathon (Table 1). Uridine is a nucleoside formed via the conjugation product of ribose and uracil and is a key component in RNA and subsequently protein synthesis^22^. This, in conjunction with the reduced ribose, indicated possible increased nucleotide production via the activation of the pyrimidine salvation metabolism. The pyrimidine salvation metabolism refers to the adaptive mechanism by which nucleotides can be synthesised using pre-formed nucleosides and nucleobases, usually following a nucleotide degradation period^5,22^, such as the extensive protein catabolism induced by a marathon^6,7,10^. These recycled nucleotides can also interact with lipids to form pyrimidine nucleotide–lipid conjugates, a key component in cell membrane structures^22^. Considering this, the slight reduction in uridine concentrations observed 24 h after the completion of the marathon, may also be associated with the restoration of damaged cellular membranes, which occur as a result of strenuous exercise^10^. This is further substantiated by the reduction/recovery of myo-inositol concentrations within 24 h post-marathon, as it is a well-known constituent of glycerophospholipids^23^.

Additionally, the de novo pyrimidine synthesis pathway is dependent on various elements of the pentose phosphate pathway^22^, particularly ribose-5-phosphate and phosphoribosyl pyrophosphate^24^. Since these are interlinking substrates for the production of erythrose-4-phosphate^24^, the recovery of erythritol (produced from the latter)^25^ not only suggests the activation of glycogenesis via the utilisation of pentose phosphate pathway intermediates, but further supports the activation of the abovementioned nucleotide repair mechanisms. Furthermore, since the majority of the erythritol is absorbed rather than metabolized^26^, as well as the fact that it is a well-known alternative sugar added to low-calorie foods^27^ frequently ingested by athletes, the alterations of this metabolite 24 h and 48 h post-marathon may also be due to reduced ingestion of such foods by the athletes during the recovery period. Similarly, several of the metabolites in Table 1, including mannitol, psicose, sorbose^27,28^, tagatose^29^, and xylose^24^, are natural constituents of fruit and vegetables, Food and Drug Administration approved alternative bulk sweeteners, and/or the products of metabolised sweeteners such as threonic acid^30^, and arabitol via arabinose^31^. Considering that endurance races reportedly alter gut microbiome composition, as well as increase intestinal permeability resulting in gut microbiome (GM) metabolite migration^32^, reductions in several of the detected metabolite markers (Table 1)^31,33^ could also be ascribed to the restoration of GM homeostasis and a reduction in host substrate availability/dietary ingestion.

### Lipid metabolism

The aforementioned depletion of intracellular glucose and glycogen stores results in extensive lipolysis of triacylglycerols in adipose tissue, skeletal muscle and the liver during strenuous activity^5,10,34^. Reduced lipolysis intermediate (α-linolenic acid, 11,14-eicosadienoic acid, 11-eicosenoic acid, 5-dodecenoic acid, docosahexaenoic acid, eicosapentaenoic acid, lauric acid, myristoleic acid, oleic acid, palmitic acid, palmitoleic acid, glycerol, and glycerol monopalmitic acid) concentrations were observed within 24 h post-marathon (Table 1) and coincided with similar findings from previous literature^5–7^. The normalisation of these is a clear attestation of the down-regulation of lipolysis^7^ mainly due to the aforementioned restoration of the glycaemic flux, concurrent with reduced ATP requirements during passive recovery^34,35^. The initial rapid reduction in serum fatty acids within 24 h post-marathon followed by a slight increase after 48 h post-marathon (Table 1) suggests a temporary upregulation of fatty acid oxidation, succeeded by a short period of fatty acid re-esterification^7,36,37^. According to Egan and Zierath^20^, myocellular glycogen replenishment is preferentially activated during the first 24–48 h post-exercise, subsequently causing a temporary increase in fat oxidation during this time, thus substantiating the aforementioned. Withal, normalised concentrations of β-hydroxyhexanoic acid (a β-oxidation intermediate) within the 24 h post-marathon recovery period indicates the restoration of the previously mentioned β-oxidation and redox imbalances^10,38^, subsequently eliminating the need for alternative fatty acid catabolism pathways, such as α-oxidation, which was previously proposed to be up-regulated during strenuous exercise^10^. This hypothesis is further supported by a reduction in α-hydroxyoctanoic acid (an α-oxidation intermediate) and the odd-chain fatty acids (OCFAs; tridecanoic acid, pentadecanoic acid, heptadecanoic acid, and 10-heptadecenoic acid). However, it should be noted that reductions in OCFAs, as well as some of the aforementioned even-chain fatty acids, may also be ascribed to alleviated autophagy^39^, reduced dietary ingestion of these, and an altered microbial activity^40^.

### Ketone production and the TCA cycle

The apparent reduced need for ATP post-marathon^35^ and the subsequently reduced fuel substrate catabolism during passive recovery is anticipated to cause a reduction in both acetyl-CoA and malonyl-CoA synthesis^34^. The recovery of the malonic acid concentrations within 24 h post-marathon not only substantiates the latter as it coincides with the previously mentioned transient increase in long-chain fatty acid oxidation, but could also be an indication of said elevated fatty acid synthesis. Furthermore, the restored concentrations of TCA cycle intermediates (α-ketoglutaric acid, fumaric acid, citric acid, and malic acid) and ketones (acetoacetic acid and β-hydroxybutyric acid) to pre-marathon concentrations within 24 h after the marathon (Table 1), support the aforementioned glycaemic flux recovery. This concurs with Nieman et al.^7^, who reported that these pathways remain perturbated for approximately 14 h post-exercise. Additionally, reduced concentrations of ketones, particularly β-hydroxybutyric acid, support the restoration of the previously mentioned redox imbalance^10,38^ and suggest an enhanced metabolic clearance rate^41^ in athletes within 24 h post-marathon. β-Hydroxybutyric acid is also a signalling molecule serving as a regulator of gene expression and adaptive responses via the inhibition of class I histone deacetylases, rendering this metabolite invaluable to the recovery process^41^.

### Amino acid metabolism

In accordance with the carbohydrates and lipids profile, various amino acids (alanine, aspartic acid, glycine, glutamic acid, methionine, phenylalanine, and tyrosine) and associated metabolites (glutaric acid, *p*-hydroxyphenylacetic acid, *p*-hydroxyphenyllactic acid, pyroglutamic acid, and hydroxyproline) recovered to baseline concentrations within 24 h after the completion of the marathon (Table 1), indicating a reduced need for amino acid catabolism during this recovery phase. However, the slight reductions in these amino acids that occur between 24 and 48 h of recovery could be an indication of protein synthesis, as substantiated by the aforementioned uridine/ribose profile (Table 1). Contrarily, slight elevations in recovery time-point hydroxyproline may be indicative of delayed post-marathon collagen catabolism, thus supporting previous literature^42^. Furthermore, the reduced concentrations of α-hydroxybutyric acid (a threonine/methionine metabolism intermediate) within 24 h post-marathon compared to the elevated post-marathon (Day 0) profile correlates with reduced amino acid catabolism and further suggests recovery of previously reported transiently hampered insulin secretion^10^, glucose absorption, and imbalance redox potential^43^.

In addition to the proteinogenic amino acids, elevated concentrations of the branched-chain amino acid (BCAA) valine, in conjunction with reduced BCAA-associated catabolism intermediates (Table 1) such as β-hydroxyisobutyric acid (valine metabolism), α-ethylhydracrylic acid (allo-isoleucine metabolism), and β-hydroxyisovaleric acid (leucine metabolism), are indicative of a reduction in BCAA catabolism. BCAAs are reportedly one of the major factors regulating mammalian target of rapamycin complex 1 (mTOR1), a multi-protein complex responsible for the activation of numerous biological processes imperative to metabolic recovery, including cell proliferation, gene expression for lipid and protein synthesis, as well as the inhibition of cellular autophagy^44^. The proposed activation of these recovery mechanisms is further substantiated by the aforementioned uridine/ribose profile (Table 1), as well as the reduction in various cell membrane-associated intermediates, such as fatty acids (particularly OCFAs), myo-inositol and cholesterol intermediates (squalene and 5-pregnen-3β,20α-diol) observed within 24 h post-marathon. Reduced concentrations of cholesterol intermediates within 24 h after the marathon in comparison to the post-marathon (day 0) concentrations also suggest inactivation of steroidogenesis^45^, which was previously proposed to be induced as a result of endurance activity^10,37^.

Aminomalonic acid was also observed to recover to pre-marathon concentrations within 24 h post-marathon. Although this metabolite has been associated with oxidative damage to protein/amino acid residues^46^, it has also been isolated from *Escherichia coli*^47^. As such, its precise mechanism of action pertaining to the metabolic recovery process remains to be elucidated.

## Conclusion

The results of this investigation suggest that the serum metabolome of athletes largely recovers to baseline (pre-marathon) status within 24 h, with total recovery evident within 48 h post-marathon without the intervention of recovery aids. This can be ascribed to a reduction in ATP requirements during passive recovery, which in turn allows for the activation of anabolic recovery mechanisms or pathways. The possible cascade of events resulting in total metabolome recovery within 24/48 h post-marathon are: (a) reduced energy requirements promoting the restoration of the imbalanced redox potential and intracellular glycaemic flux, thus stimulating glycogenesis. (b) In turn, this further reduces the need for alternative fuel substrate catabolism (of lipids and amino acids) and ketogenesis, (c) activating additional cellular repair mechanisms such as the uridine-dependent nucleotide salvage pathway, mTOR1 stimulation (subsequently inactivating cellular autophagy), as well as possible protein and lipid synthesis/fatty acid re-esterification. Moreover, GM-associated markers emphasize the close metabolic interaction between man and microbe.

## Limitations and future prospects

Possible limitations of this investigation may include the inevitable confounder of human genotype/phenotype (sex, age and ethnicity) variation, as well as individual athlete dietary variation. Although the athletes’ dietary intake was recorded throughout the investigation, adding dietary restrictions to the already refrained use of recovery aids would be considered too much of an interference to the personalised regimens of these athletes. However, in an attempt to circumvent the variability in participant demographics (age, sex, fitness, running experience, etc.), the study design included repeated measures of the same individuals and the use of statistical methods that consider the dependence between observations (therefore each participant becomes their own control)^48,49^. Nevertheless, such individual variation allows for a higher level of robustness in the results, which provides a better representation of the true nature of the activated recovery mechanisms as the body attempts to reach homeostasis.

Although the current investigation provided a holistic (untargeted) representation of the metabolome recovery after a marathon, future studies may consider applying more targeted approaches based on these results to possibly identify more personalised/efficient recovery modalities. Additionally, since this investigation was performed on a relatively small cohort, future investigations consisting of larger cohorts and/or serum samples obtained at additional time-points (e.g. 12 h, 36 h, and 72 h post-marathon) are required to validate these findings, ultimately allowing for better metabolic mapping of the in-between stages via the identification of additional metabolite markers pertinent to the metabolic recovery trend of endurance athletes. Considering that metabolic differences correlating with gender heterogeneity could not be positively annotated due to limited cohort size, future studies may consider including larger gender-specific cohorts. Lastly, since GM metabolism seems to be an integral part of athletic performance, more targeted analysis of the associated metabolites or using an alternative biological medium (such as faecal matter, urine, etc.) could provide a more perspicuous understanding of the importance of the GM in terms of metabolome recovery.

## Methods and materials

### Participants and the marathon race

The participants (n = 16; 10 males and 6 females) were selected at random from the pool of athletes competing in the Druridge Bay Marathon in Newcastle (UK). The eligibility of the selected participants was assessed by means of a health and dietary questionnaire, after which those individuals with food allergies, cardiovascular complications, musculoskeletal disorders/injuries, or those receiving anti-inflammatory treatment were excluded from the study. In addition, female participants completed a menstruation cycle questionnaire. Considering the ethical implications concerning ethnicity classifications of participants when it is not central to the research topic, as well as the fact that this study was not biased towards any ethnic groups, nor were any of the aims a comparison of ethnicity with respect to the marathon induced changes to the human metabolome, athlete ethnicity was not recorded. All athletes were instructed to abstain from using any recovery modalities/therapies such as cryotherapy, pressure garments, foam rolling, active recovery (moderate exercise bouts), NSAID, vitamins, etc. during the recovery period of this investigation. A summary of participant characteristics is presented in Table 2, while a summary of age, sex, and overall performance distribution of the athletes are presented in Table S2 (Supplementary information). The Druridge marathon entails running four laps around the Country Park (Northumberland coast, UK), which mainly consists of grassy, concrete mixed terrain. Approximately 1.6 km of each lap consists of soft sand (± 6.4 km)^50^ and may contribute to slower finishing times (Table 2).

**Table 2:** Participant demographical information and endurance race experience history.

Participant demographical information	Average ± standard deviation
Age (years)	39 ± 12
Pre-marathon athlete weight (kg)	72.2 ± 11.9
Post-marathon athlete weight (kg)	70.8 ± 11.7
Running experience (years)	8 ± 7
Marathon experience (races)	21 ± 40
Finishing time (hh:mm:ss)	04:30:25 ± 00:36:48

### Sample collection

This investigation forms part of a larger, double-blinded placebo study (Supplementary Fig. S2), in which 31 athletes participating in the 2016 Druridge Bay Marathon either received multiple beetroot (n = 15 athletes) or placebo (n = 16 athletes) supplements, over a period of 48 h. Blood samples (n = 124 samples) were collected 24 h before (as a fasting, yet hydrated, baseline/pre-marathon), immediately after (within 30 min; post-marathon [day 0]), as well as 24 h (day 1 post-marathon) and 48 h (day 2 post-marathon) post-marathon by means of antecubital (basilica vein) venepuncture^10,50^. Venous sampling was limited to only four sampling intervals considering the impact and inconvenience of increased blood sampling, which would have led to increased participant drop-out. These samples were procured from the Faculty of Health and Life Sciences, Department of Sport, Exercise and Rehabilitation at Northumbria University in Newcastle upon Tyne (UK), and transported to the Laboratory of Infectious and Acquired Diseases, Focus Area: Human Metabolomics at the North-West University in Potchefstroom, as previously described by Stander et al.^10^. Considering the aim of the current investigation, only the serum samples (n = 64) of the 16 athletes that ingested placebo supplements were included in this study. Furthermore, since this study only focuses on the metabolic adaptations presented during a 48 h recovery period, information regarding additional clinical serum measurements are not included, but are available in a complementary physiological publication based on the same cohort^50^.

### Sample extraction and derivatisation

As previously described in detail by Stander et al.^10^, all samples along with pooled quality control (QC) samples (pooled 50 μL of each sample) were subjected to a total metabolome extraction. As part of this process, smaller aliquots of the samples (50 μL) were prepared, before adding 50 μL of the internal standard (3-phenylbutyric acid; 0.45 μg/mL) dissolved in a chloroform:methanol:water (1:3:1) solution. The latter serves as a metabolite conservation step to ensure that larger polar and non-polar compounds and associated metabolites (e.g. amino acids, fatty acids, purines, pyrimidines, alkenes, alkanes etc.) can be analysed using the GCxGC-TOFMS. Protein precipitation of the samples commenced by adding 300 μL of ice-cold acetonitrile, where after each vial was subjected to a 2 min mixing step (REAX D-91126 vortex; Heidolph Instruments GmbH & Co.KG, Schwabach, Germany) and 10 min centrifugation at 4,000 rpm. The supernatants were extracted, transferred to a clean GC–MS vial, and dried with a light stream of nitrogen gas for 45 min, whilst being housed in a heating block set at 40 °C. Once the solvent evaporated, 25 μL of methoxamine hydrochloride dissolved in pyridine (15 mg/mL) was added to each sample and incubated for 90 min at 50 °C. Lastly, 40 μL of BSTFA enriched with 1% TCMS was added, and samples were left to derivatise/silylate for 60 min at 60 °C. Derivatised samples were then transferred to a clean glass GC–MS vial containing a vial insert.

### GCxGC-TOFMS analysis

Samples were injected into the Pegasus 4D GCxGC-TOFMS analytical apparatus (LECO Africa (Pty) Ltd, Johannesburg, South Africa) in a randomised order by using a Gerstel 104 auto-sampler tray (GmbH and co. KG, Mülheim van der Ruhr, Germany), with QC samples preceding approximately every 7th sample for quality assurance purposes. Each sample (1 μL) was injected using a 1:3 split ratio with a constant injector temperature of 270 °C and a carrier gas (purified helium) flow rate of 1 mL/min. The primary oven containing a Restek Rxi-5MS capillary column (30 m; 0.25 μm diameter and 0.25 μm film thickness) was set to operate at an initial temperature of 70 °C before incrementally (4 °C/min) increasing to a final temperature of 300 °C (maintained for 2 min). In addition, the secondary oven, fitted with a Restek Rxi-17 capillary column (1 m; 0.25 μm diameter and 0.25 μm film thickness), was programmed with an initial temperature of 85 °C which was set to incrementally increase with 4.5 °C/min until a final temperature of 300 °C was reached. The thermal modulator, amid the two columns, was set to intermittently pulse streams of hot and cold nitrogen gas for approximately 0.5 s (every 3 s). Mass spectra (ms) of ions (50–800 *m*/*z* range) was acquired at a rate of 200 ms/s, while the ms of the first 400 s of the run was discarded to exclude extraction solvent elution. However, the latter was not removed from the time axis of the primary column to ensure compound retention time validity. The transfer line and ion source were set to function at 270 °C and 220 °C, respectively, with a detector voltage of 1,600 V and ion filament bias of − 70e V. After analytical analysis, the data generated was processed using the ChromaTOF software version 4.7.1 (LECO Corporation), which included ms deconvolution, peak alignment and peak annotation via mass fragment pattern and retention time comparison with in-house libraries compiled from previously injected standards^51^. ChromaTof parameter settings included peak widths of 9 and 0.2 for the 1st and 2nd dimension respectively, and a signal-to-noise ratio of 100, with a maximum of 100,000 unknown peaks allowed (to remove bias). All metabolites with molecular masses between 50 and 800 Da were detected with a minimum of 70% similarity match to the in-house libraries for identification. The experimental procedure performed was in accordance with proposed guidelines and regulations, as approved by the North-West University (South Africa) and forms part of the laboratory’s standard operating procedures (SOP number: HM-MET-056).

### Statistical analysis and group comparison

Prior to data analysis, pooled QC samples were compared to ensure data validity before interpretation. Data analyses commenced with compound normalisation in relation to an internal standard and all identified analytical contaminants/plasticizers/column related metabolites were removed. The data was then subjected to numerous data clean-up steps, including a QC-CV filter (retaining metabolites with CV ≤ 30), QC drift/batch correction (using a pooled QC sample), zero-value replacement (with random, small values along the tail distribution of the data), 50% zero value filter, log transformation and auto-scaling^48,52–54^. Hereafter the data was subjected to a multi-statistical approach consisting of two main objectives i.e. (1) obtain a global pattern of the metabolic profile of athletes over time and to some extent validate the design against the logical result, as well as (2) determine specific metabolic adaptations between time-points (Fig. 1) to distinguish between sources of variation due to early (day 1 vs day 0) and late (day 2 vs day 0) stages recovery; from full recovery (day 2 vs pre-marathon). As such, statistical analyses consisted of both paired univariate and multivariate approaches, using MATLAB^55^ with a PLS toolbox^56^. In terms of the multivariate analyses, an ML-PCA were used to inspect specific time-point differences in the multivariate space, whereas and an ASCA was employed to summarise the overall metabolic perturbation observed over the entire timeframe of the study^57^. Both these methods take the dependence between observations into account. Furthermore, univariate data analyses included a paired *t*-test, corrected for multiple testing by controlling the FDR (limiting to 5%) using the Benjamini–Hochberg procedure^58^, and a dependent effect size test to respectively assess the statistical and practical significance of each metabolite^48^.

Considering the inevitable confounder of inter-individual variation, a repeated measures statistical design was employed to control for the latter as each athlete’s metabolic variation is evaluated relative to him/herself. That said, statistical approaches for paired data may not correct for all confounders and as such this design aimed to ensure equal representation across different confounders as indicated in Table S2 (Supplementary information). Although more complex models were considered for variable selection (i.e. repeated measures ANOVA), these models would not be applicable due to the small sample size and were therefore excluded.

### Ethical approval

Ethical approval for this investigation, conducted according to the Declaration of Helsinki and International Conference on Harmonization Guidelines, was obtained from the Research Ethics Committee of the Faculty of Health and Life Sciences at the Northumbria University in Newcastle upon Tyne, UK (reference number: HLSTC120716). Informed, written consent was obtained from all individuals included in the study.

## Supplementary information

Supplementary file 1 (PDF 420 kb)

Supplementary file 2 (XLSX 23 kb)

## Data Availability

As the current investigation is part of a larger collaboration study consisting of multiple aims, the dataset of this investigation is not yet publicly available but can be acquired from the corresponding author on reasonable request. The authors declare that all the results included in this study have been presented clearly, honestly and without fabrication, falsification, or inappropriate data manipulation.
