# Supplementary material for: The unaided recovery of marathon-induced serum metabolome alterations
Source: Sci Rep. 2020 Jul 6;10:11060. doi: 10.1038/s41598-020-67884-9 (PMC7338546; doi:10.1038/s41598-020-67884-9)
Supplement: Supplementary file 1 — (PDF 420 kb) [file 41598_2020_67884_MOESM1_ESM.pdf]

# SUPPLEMENTARY INFORMATION

# The unaided recovery of marathon-induced serum metabolome alterations

**Abbreviated title:** Post-marathon metabolic recovery

Zinandré Stander[1], Laneke Luies[1], Lodewyk J Mienie[1], Mari van Reenen[1], Glyn Howatson[2,3], Karen M Keane[2], Tom Clifford[4,5], Emma J Stevenson[4], Du Toit Loots[1*]

*[1]Human Metabolomics, Faculty of Natural and Agricultural Sciences, North-West University, Potchefstroom, South Africa, 2531; [2]Faculty of Health and Life Sciences, Department of Sport, Exercise and Rehabilitation, Northumbria University, Newcastle upon Tyne, United Kingdom, NE1 8ST; [3]Water Research Group, School of Environmental Sciences and Development, North-West University, Potchefstroom, South Africa, 2531; [4]Human Nutrition Research Centre, Institute of Cellular Medicine, Newcastle University, Newcastle upon Tyne; [5]School of Sport, Exercise and Health Sciences, Loughborough University, United Kingdom.*

*Corresponding author: Prof. Du Toit Loots

E-mail: dutoit.loots@nwu.ac.za; ORCID: 0000-0002-0339-6237

Contact: +27 (0) 18 299 1818; Fax: +27 (0) 18 299 1823

Postal address: North-West University, Potchefstroom Campus, Private Bag X6001, Box 269, Potchefstroom, 2520.

Mrs Zinandré Stander: 25098365@nwu.ac.za; ORCID: 0000-0001-8281-5112

Dr Laneke Luies: laneke.luies@nwu.ac.za; ORCID: 0000-0003-4920-4090

Dr Mari van Reenen: 12791733@nwu.ac.za; ORCID: 0000-0002-5856-3258

Prof. Japie Mienie: japie.mienie@nwu.ac.za; ORCID: 0000-0003-0860-5285

Prof. Glyn Howatson: glyn.howatson@northumbria.ac.uk; ORCID: 0000-0001-8494-2043

Dr. Tom Clifford: tom.clifford@newcastle.ac.uk; ORCID: 0000-0003-0484-2953

Prof. Emma Stevenson emma.stevenson@newcastel.ac.uk; ORCID:0000-0001-9388-3903

Dr Karen Keane k.keane@northumbria.ac.uk; ORCID: 0000-0002-1572-9211

# 1. SUPPLEMANTRARY INFORMATION

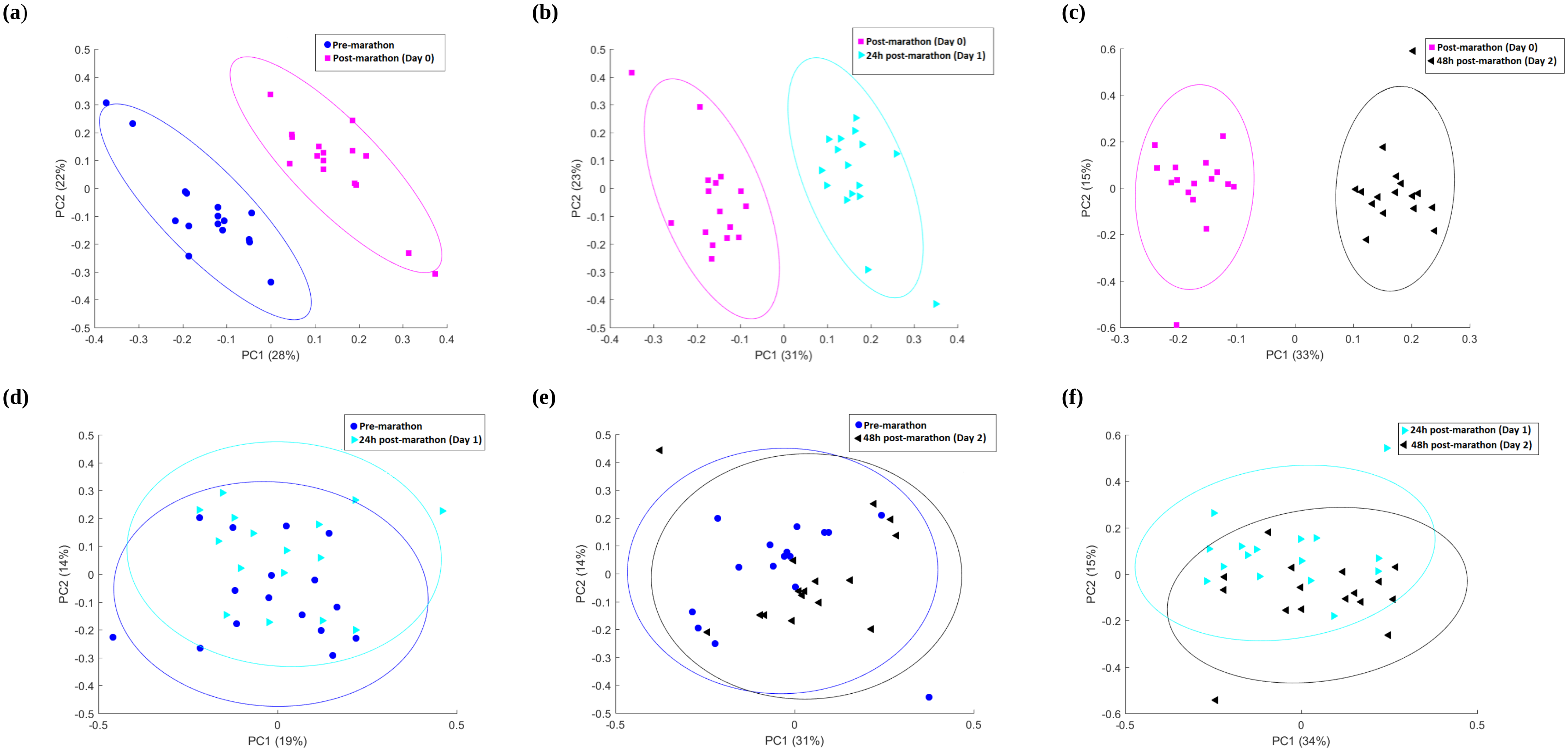


***Fig. S1*** *Multilevel principle component analysis plots of the comparative groups: (a) Pre-marathon (denoted by blue/circle) vs post-marathon (denoted by pink/square); (b) post-marathon (denoted by pink/square) vs 24 h post-marathon (denoted by turquoise/triangle); (c) post-marathon (denoted by pink/square) vs 48 h post-marathon (denoted by black/triangle); (d) pre-marathon (denoted by blue/circle) vs 24 h post-marathon (denoted by turquoise/triangle); (e) pre-marathon (denoted by blue/circle) vs*

*48 h post-marathon (denoted by black/triangle); (f) 24 h post-marathon (denoted by turquoise/right-tilted triangle) vs 48 h post-marathon (denoted by black/left-tilted triangle). Abbreviations: PC: principle component.*

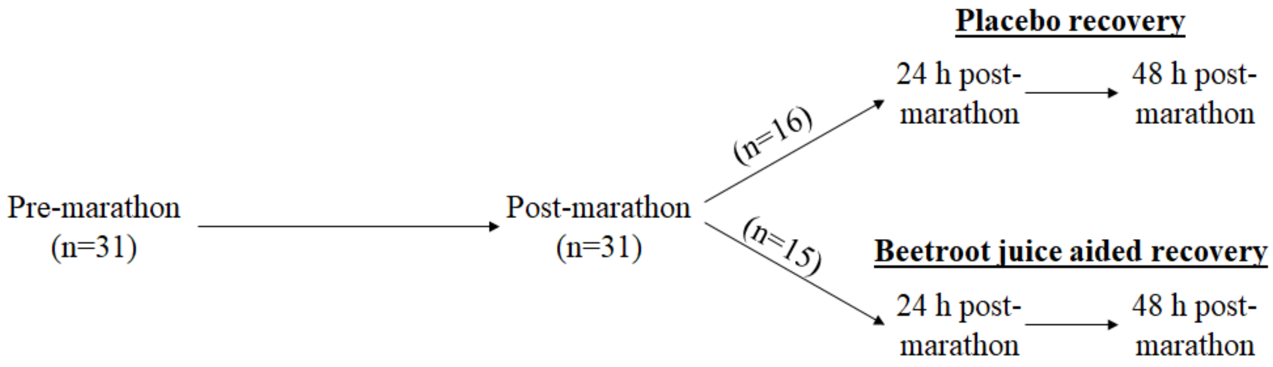


***Fig. S2*** *The larger scope of this investigation consists of multiple objectives: Objective 1: Effects of a marathon on the serum metabolome of athletes (pre- vs post-marathon; n=31) [10]; Objective 2 (current manuscript objective): Metabolic recovery without the intervention of recovery aids, by comparing pre-, post- as well as 24 h and 48 h post-marathon samples of the athletes that ingested placebo supplements (n=16); Objective 3: Effect of beetroot juice supplementation on metabolic recovery, by comparing pre-, post- as well as 24 h and 48 h post-marathon samples of the athletes that ingested beetroot juice supplements (n=15).*

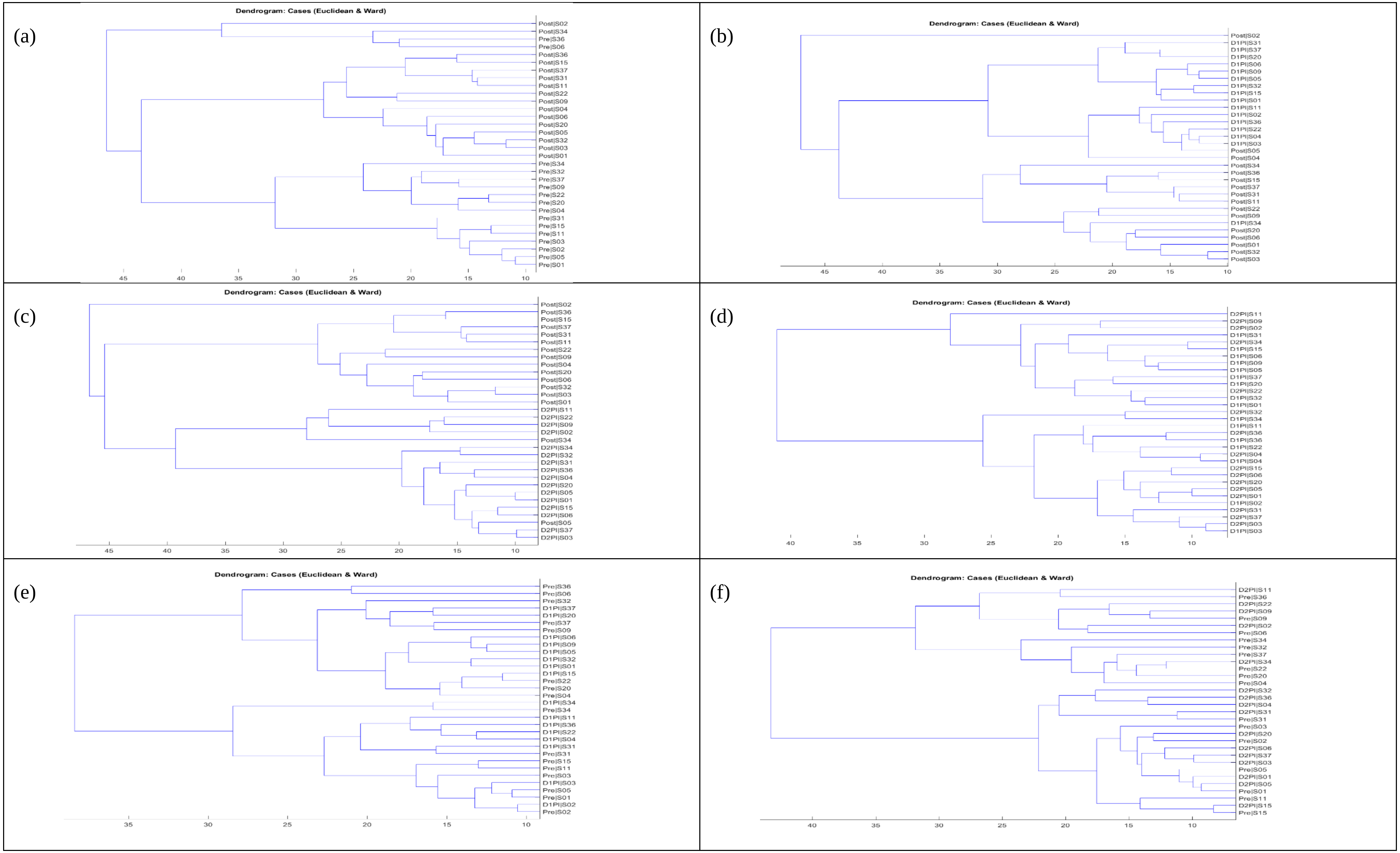


***Fig. S3:*** *Dendrogram metabolome clustering of athletes at the various comparative timepoints: (a) Pre-marathon vs post-marathon, (b) Post-marathon vs 24 h post-marathon, (c) Post-marathon vs 48 h post-marathon, (d) 24 h vs 48 h post-marathon, (e) Pre-marathon vs 24 h post-marathon, and (f) Pre-marathon vs 48 h post-marathon.*

***Table S2:*** *Participant characteristics and performance-distribution of the entire cohort.*

| **Distribution count and frequency** | **Criteria** | | | | | | |
|---|---|---|---|---|---|---|---|
| **Age distribution** *(yrs)* | | | | | | | |
| | **20-30** | **30-40** | **40-50** | **50-60** | **60-70** | | |
| Count | 3 | 4 | 7 | 0 | 2 | | |
| Frequency | 18.7% | 25.0% | 43.7% | 0.0% | 12.5% | | |
| **Gender** | | | | | | | |
| | **Female** | **Male** | | | | | |
| Count | 6 | 10 | | | | | |
| Frequency | 37.5% | 62.5% | | | | | |
| **Marathon performance** *(duration)* | | | | | | | |
| | **3h00–3h30** | **3h30–4h00** | **4h00–4h30** | **4h30–5h00** | **5h00–5h30** | **5h30–6h00** | **6h00–6h30** |
| Count | 2 | 5 | 2 | 2 | 2 | 2 | 1 |
| Frequency | 12.5% | 31.2% | 12.5% | 12.5% | 12.5% | 12.5% | 6.2% |
